# Fueling or Fighting Cancer? The Thermogenic Paradox of Brown Adipose Tissue

**DOI:** 10.1007/s13679-026-00699-3

**Published:** 2026-03-18

**Authors:** Jonathan Jaime G. Guerrero, Paolo C. Encarnacion, Chih-Hao Wang, Mark Angelo S. del Rosario, Kin Israel Notarte, Jiayan Zhou, Yi-Ta Hsieh, Wan-Yu Wang, Ching-Wen Chang, Wan-Chun Li

**Affiliations:** 1 College of Medicine, University of the Philippines, Manila, Philippines; 2 College of Public Health, University of the Philippines, Manila, Philippines; 3 Genomics Research Center, Academia Sinica, Taipei, Taiwan; 4 Department of Pathology, Johns Hopkins University School of Medicine, Baltimore, MD USA; 5 Department of Medicine, Stanford University School of Medicine, Stanford, CA USA; 6 Department of Nursing, Min-Hwei College of Health Care Management, Tainan, Taiwan; 7 Division of General Surgery, Department of Surgery, Shuang Ho Hospital, Taipei Medical University, New Taipei City, Taiwan; 8 Graduate Institute of Metabolism and Obesity Sciences, Taipei Medical University, Taipei, Taiwan; 9 TMU Research Center for Digestive Medicine, Taipei Medical University, Taipei, Taiwan; 10 Taipei Cancer Center, Taipei Medical University, Taipei, Taiwan; 11 Institute of Oral Biology, College of Dentistry, National Yang Ming Chiao Tung University, Taipei, Taiwan; 12 Department of Dentistry, College of Dentistry, National Yang Ming Chiao Tung University, Taipei, Taiwan; 13 Oral Medicine Innovation Center (OMIC), National Yang Ming Chiao Tung University, Taipei, Taiwan; 14 Department of Stomatology, Taipei Veterans General Hospital, Taipei, Taiwan

**Keywords:** Adipose browning, Cancer-associated cachexia, Thermogenesis, Metabolic reprogramming, Uncoupling protein 1, Adipokine signaling

## Abstract

**Purpose of Review:**

The global rise in obesity and metabolic syndrome has intensified interest in brown adipose tissue (BAT) as a regulator of energy metabolism and potential modulator of cancer risk. BAT-mediated thermogenesis and the browning of white adipose tissue (WAT) confer metabolic benefits that may reduce oncogenic susceptibility. However, emerging evidence reveals a paradoxical role for BAT in cancer progression, where tumor-induced thermogenic activation contributes to cancer-associated cachexia (CAC). This review article examines the cellular, molecular, and translational dimensions of this “thermogenic paradox”.

**Recent Findings:**

A narrative synthesis was performed using literature from 2000 to 2025 retrieved from PubMed, Scopus, Web of Science, and Google Scholar. Peer-reviewed studies examining the molecular, genetic, and metabolic mechanisms linking BAT or adipose browning to carcinogenesis, obesity-related cancers, and CAC were included. Thematic integration emphasized regulatory pathways, endocrine signaling, and therapeutic implications. Adaptive browning, regulated by transcriptional drivers such as PRDM16, PPARγ, and PGC1-α, mitigates metabolic inflammation, enhances insulin sensitivity, and may exert tumor-suppressive effects. In contrast, tumor-secreted factors including parathyroid hormone-related protein (PTHrP) and interleukin-6 aberrantly induce uncoupling protein 1 (UCP1) expression and β3-adrenergic signaling, driving lipolysis and energy wasting in CAC. The dualistic effects of BAT underscore its context-dependent influence on cancer biology.

**Summary:**

BAT exemplifies a metabolic continuum between protection and pathology. Clarifying its regulatory mechanisms may inform precision therapies and integrated metabolic-oncology interventions, particularly relevant to low- and middle-income countries facing the double burden of obesity and cachexia.

**Graphical Abstract:**

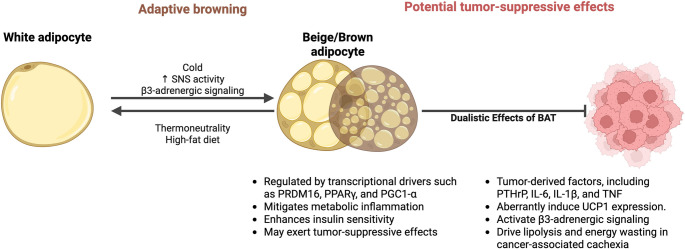

## Introduction

The Global cancer burden must be viewed through the lens of the escalating metabolic health crisis. Driven by the soaring prevalence of obesity and metabolic syndrome, nearly three billion people worldwide will be overweight or obese by 2035 [1]. It is well known that obesity and related metabolic disorders, characterized by severe energy imbalance and excess visceral fat accumulation, are potent drivers of carcinogenesis [2–5]. Metabolic dysfunction significantly contributes to the global cancer burden, with high body mass index (BMI) accounting for a significant proportion of adult deaths from Noncommunicable Diseases (NCDs) each year [6, 7]. The contribution of excess body weight to cancer incidence is significant and quantifiable, reaching as high as 10.6% of new cancer cases in women aged 30 and older in the United States in 2019 [8]. This epidemiological risk also exhibits regional heterogeneity: for instance, the Asia-Pacific region reports stronger associations between obesity and both premenopausal and postmenopausal breast cancer [9, 10], and in 2021, Mongolia recorded the world’s highest age-standardized death rate of liver cancer, much of which is attributed to high BMI [11].

Collectively, these global epidemiological patterns point to a unifying biological framework linking metabolic dysfunction to cancer development. The consistency and magnitude of obesity-associated cancer risk across diverse populations indicate that excess adiposity operates not merely as a contextual risk factor, but as an active biological driver of tumor initiation and progression. This perspective elevates obesity from a comorbidity to a central contributor to oncogenesis and underscores the need to interrogate adipose tissue biology as the critical interface through which the global metabolic health crisis translates into cancer burden [2–5].

Adipose tissue is now understood to function as an active endocrine organ that releases signaling molecules called adipokines [12]. In obesity, this pathogenic state, termed “adiposopathy,” creates a pro-tumorigenic environment where most secreted adipokines enhance cell proliferation, migration, and anti-apoptosis pathways [13, 14]. Key to this dysregulation is the balance between Leptin and Adiponectin [15]. Leptin, often increased in obesity, acts as a pro-tumorigenic signal, driving malignancy by activating the epithelial–mesenchymal transition (EMT) program, promoting invasion through Matrix Metalloproteinase (MMP) upregulation, and stimulating pro-tumorigenic M2 macrophages [15–17]. Adiponectin generally exhibits an inverse relationship with cancer risk; however its role is context-dependent, as it may paradoxically promote tumor progression in specific microenvironments by enhancing angiogenesis or inducing stromal fibroblast senescence [14, 18, 19]. Given that obesity is a modifiable risk factor, its prevention and management remain a core oncological strategy [20, 21]. Importantly, pre-diagnostic obesity is a strong negative prognostic indicator, associated with decreased overall survival and increased cancer-specific mortality [22, 23].

While excess adiposity drives cancer incidence, the progression of advanced disease often presents the devastating, opposing syndrome of cancer-associated cachexia (CAC) [24]. CAC is a multifactorial syndrome defined by the severe and intractable wasting of fat and muscle tissue, which drastically reduces patient quality of life and survival [25]. Management remains challenging, with limited efficacy of available pharmacologic interventions highlighting the urgent need for novel biomarkers and analytic approaches capable of predicting early onset and monitoring disease progression [26, 27].

A central molecular feature underlying cachectic wasting is the paradoxical metabolic switch known as fat browning [3, 28]. This process involves the activation of Brown adipose tissue (BAT) and the conversion of white adipocytes into thermogenic beige fat, which is driven by the high-level expression of mitochondrial uncoupling protein 1 (UCP1) [29, 30]. While UCP1-mediated thermogenesis is a beneficial strategy for combating obesity and metabolic syndromes by increasing energy expenditure and promoting metabolic stability [31], tumor-derived factors drive the pathological hyperactivation of UCP1 [32, 33]. This excessive thermogenesis, coupled with increased white adipose tissue lipolysis, results in an uncompensated state of high energy expenditure, which directly drives a negative energy balance and systemic wasting in cachexia [34]. This metabolic switch is the key determinant connecting metabolic risk to oncological wasting.

For clarity, this review distinguishes between classical BAT and adipose browning. BAT refers to developmentally distinct depots of thermogenic adipocytes, most abundant in infancy but metabolically active in adults. In contrast, adipose browning describes the inducible emergence of beige adipocytes within white fat depots under stimuli such as cold exposure, exercise, or tumor‑derived factors [35–37]. While both BAT and beige adipocytes share thermogenic machinery (e.g. UCP1 mediated effects), their developmental origins, regulatory pathways, and physiological roles differ. Throughout this article, we use “BAT” to denote classical brown depots and “adipose browning” to denote the inducible beige phenotype [38].

Given the complex and often contradictory findings, a comprehensive examination of the molecular mechanisms governing adipose browning is critically needed. This need is particularly acute within a global health equity framework, as low- and middle-income countries disproportionally bear the dual burden of obesity-related cancer and cachexia [39–41]. This review aims to detail the current molecular, cellular, and genetic mechanisms underlying the role of adipose browning in cancer progression and cachexia, thereby informing future translational strategies.

## Methods

This review was conducted as a narrative synthesis of the available evidence on the role of BAT or adipose browning in carcinogenesis, obesity-associated malignancies, and CAC. The methodology was designed to ensure a comprehensive yet critical integration of molecular, genetic, and metabolic perspectives.

### Literature Search Strategy

A structured literature search was performed using PubMed, Scopus, Web of Science, and Google Scholar, covering studies published between 2000 and 2025. Foundational studies published prior to 2000 were also intentionally included when they provided seminal insights into BAT biology and adipose browning. Search terms combined Medical Subject Headings (MeSH) and free-text keywords, including *“brown adipose tissue*,*” “BAT*,*” “adipose browning*,*” “beige adipose*,*” “thermogenesis*,*” “carcinogenesis*,*” “cancer cachexia*,*”* and *“obesity-related cancer.”* In addition, reference lists of key articles were manually reviewed to capture studies not identified in the database search.

### Eligibility Criteria

Peer-reviewed articles published in English were eligible for inclusion, including original research articles, systematic reviews, meta-analyses, or narrative reviews. Studies included if they investigated the molecular, genetic, cellular, or metabolic mechanisms by which BAT or adipose browning influences cancer development, progression, or cachexia. Both preclinical (animal and cellular) and human studies were considered, reflecting the translational scope of the topic. Preference was given to articles that explicitly addressed the dual role of browning, either as a protective mechanism against obesity-driven carcinogenesis or as a pathological contributor to tumor-induced metabolic wasting.

Studies were excluded if they were not peer-reviewed, such as opinion pieces, editorials, or conference abstracts without full text. Articles were also excluded if they focused solely on obesity, metabolic syndrome, or energy balance without explicit connections to cancer outcomes. Case reports, anecdotal descriptions, and publications in languages other than English were similarly omitted.

### Data Extraction and Synthesis

From each included article, relevant information was extracted, including study design, model system, molecular pathways analyzed, and implications for carcinogenesis or cachexia. Findings were synthesized narratively under thematic categories: (i) adipose tissue biology and browning mechanisms, (ii) tumor-suppressive roles of BAT, (iii) tumor-promoting mechanisms in cachexia, and (iv) therapeutic and translational perspectives. Areas of consensus, controversy, and knowledge gaps were highlighted.

## Results

### Adipose Tissue Biology and Browning Phenomena

#### White, Brown, and Beige Adipose Tissue

Adipose tissue exists in three major forms with distinct metabolic roles. White adipose tissue (WAT) primarily stores energy as triglycerides and functions as an endocrine organ, secreting adipokines such as leptin and adiponectin [12]. In obesity, WAT undergoes pathological remodeling (“adiposopathy”), creating a pro‑tumorigenic environment characterized by chronic inflammation and dysregulated adipokine signaling [13, 14].

In contrast, BAT is specialized for non‑shivering thermogenesis through the mitochondrial protein UCP1 [42, 43]. BAT is highly vascularized and innervated, enabling rapid energy dissipation as heat. Though most abundant in infants, BAT remains metabolically relevant in adults [30].

Beige adipocytes emerge within WAT depots under specific stimuli, such as cold exposure or β-adrenergic signaling, and share thermogenic features with BAT despite distinct developmental origins [31]. This “browning” process enhances energy expenditure, improves insulin sensitivity, and reduces metabolic inflammation.

Collectively, these adipose phenotypes illustrate the dual metabolic potential of fat tissue: WAT remodeling fosters tumorigenesis, while BAT and beige fat confer protective metabolic effects [37, 44]. Importantly, the same thermogenic machinery that benefits metabolic health can be co-opted by tumors, driving CAC [45, 46]. This paradox underscores the need to examine browning not only as a metabolic adaptation but also as a determinant of cancer progression.

#### Lifestyle Modulation of Adipose Browning

The activation of thermogenesis leads to increased energy expenditure and the combustion of nutrients, thereby improving metabolic health parameters such as glucose homeostasis, insulin sensitivity, and hyperlipidemia [47, 48]. The induction of browning is highly sensitive to environmental and lifestyle factors. Intermittent cold exposure (ICE), for instance, consistently increases the activity of BAT and promotes the conversion of WAT to a beige phenotype, yielding positive modulation of metabolic consequences like improved glucose tolerance [49–51]. Mechanistically, exercise-induced expression of the transcriptional coactivator Peroxisome proliferator-activated receptor g coactivator1-α (PGC1-α) in muscle stimulates the release of the myokine Irisin [52, 53]. Irisin acts on white adipose cells to stimulate UCP1 expression and promote brown fat-like development, providing a critical molecular link explaining the robust anti-cancer prophylaxis associated with physical activity [47, 48, 54–56]. Even though emerging evidence indicates that both cold exposure and exercise can activate BAT, practical guidance is essential for translation into clinical or lifestyle interventions.

**Fig. 1:**
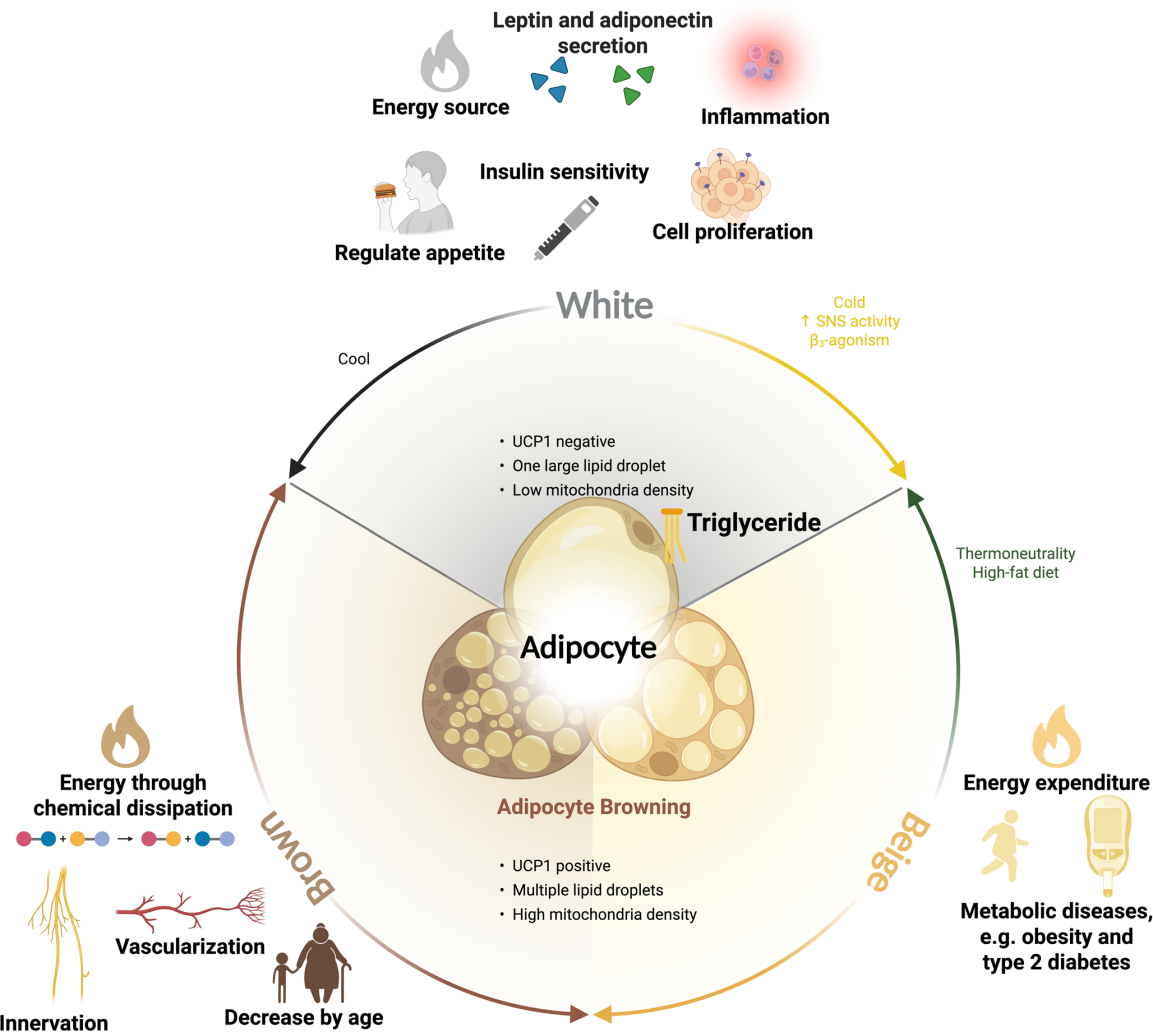
Adipocyte phenotypes and metabolic consequences of BAT activation and browning. The diagram contrasts white, beige, and brown adipocytes and summarizes how environmental cues shape their function. White adipocytes store triglycerides, are UCP1-negative, contain one large lipid droplet, and have low mitochondrial density. Cold exposure and beta-adrenergic stimulation promote browning toward a beige phenotype, which increases mitochondrial content and thermogenic capacity. Brown and beige adipocytes are UCP1-positive, contain multiple small lipid droplets, and dissipate energy as heat. Innervation and vascularization support this thermogenic program, which tends to decline with age. Thermoneutral conditions and a high-fat diet favor whitening. Adipocyte states are linked to systemic outcomes, including energy expenditure, insulin sensitivity, appetite regulation, leptin and adiponectin secretion, cell proliferation, inflammation, and risk of metabolic disease. The figure was created with BioRender.com; a publication license was obtained

Human studies demonstrated that mild cold exposure at temperatures of approximately 16–19 °C for 1–2 h per session, repeated 3–5 times per week, is sufficient to stimulate BAT activity and enhance glucose metabolism [43, 57, 58]. Chronic exposure over several weeks further augments BAT volume and thermogenic capacity. In parallel, moderate-intensity aerobic exercise, such as running, cycling, or brisk walking, for 30–60 min per session, performed 3–5 times weekly, has been consistently linked to increased BAT metabolic activity and adipose browning via endocrine mediators including irisin and Fibroblast Growth Factor-21 (FGF-21) [59, 60]. Resistance training, while beneficial for overall metabolic health, appears to exert weaker direct impacts on BAT activation, though it may complement aerobic exercise by improving insulin sensitivity [61]. Taken together, these findings suggest that a combination of regular mild cold exposure and structured aerobic exercise provides the most effective strategy for promoting BAT activity in humans. Figure 1 illustrates the phenotypic differences between white, beige, and brown adipocytes, highlighting how environmental cues such as cold exposure and exercise shape their metabolic consequences. This schematic reinforces the paradox whereby browning confers metabolic protection in obesity yet can be co-opted by tumors to drive CAC.

#### Molecular and Genetic Basis of Adipose Browning

**Fig. 2:**
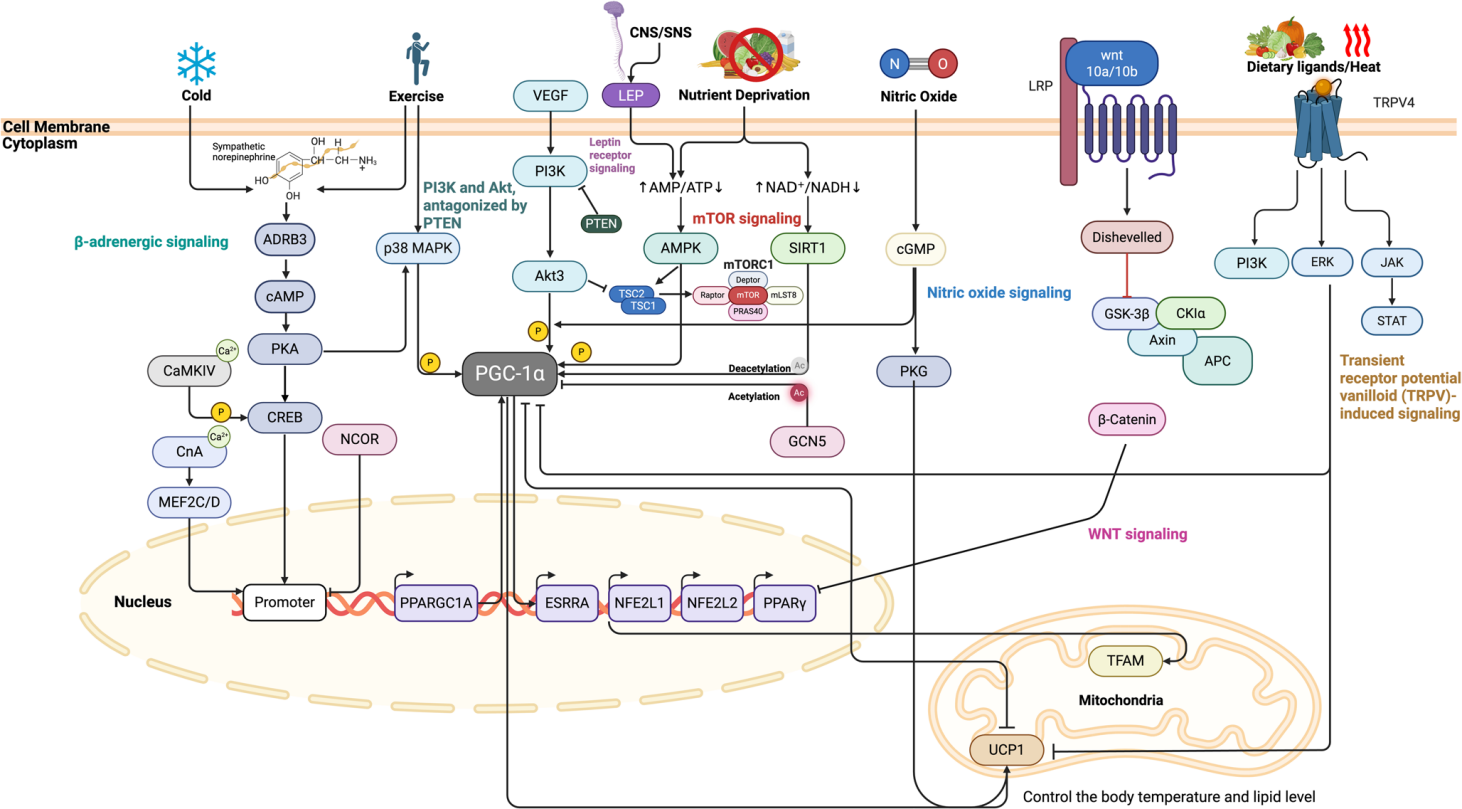
Regulatory pathways potentially involved in the promotion of browning and BAT activation in the context of cancer. Environmental and nutritional cues, such as cold exposure, exercise, nutrient deprivation, nitric oxide, and dietary signals, engage upstream sensors and receptors, including beta-adrenergic receptors, VEGF signaling, AMPK, and TRPV1. These inputs converge on canonical pathways, notably beta-adrenergic, nitric oxide, and WNT signaling, and activate the transcriptional coactivator PGC-1α through mediators such as CREB, CaMK, and SIRT1. PGC-1α partners with transcription factors including PPARGC1A, ESRRA, NRF1, NRF2, MEF2A, and PPARG to induce genes that drive mitochondrial biogenesis, thermogenesis, and lipid handling. The resulting program increases energy expenditure and supports the browning of white adipocytes. Mitochondrial remodeling is central to this process, with UCP1 serving as a key effector of thermogenesis and lipid flux. In carcinogenesis, tumor- and host-derived cues can modulate these networks, thereby influencing adipose remodeling and systemic metabolism. The figure was created with BioRender.com; a publication license was obtained

At the molecular and genetic level, adipose browning is a tightly regulated process that converts energy-storing white adipocytes into metabolically active, thermogenic cells. This transition is driven by coordinated changes in gene expression as key transcriptional regulators and signaling pathways integrate hormonal, neural, and environmental cures to activate this program. Figure 2 summarizes regulatory pathways that converge on PGC1α and UCP1 to drive browning. By integrating tumor-derived signals into these networks, the figure underscores how the same pathways beneficial in metabolic health may become maladaptive in CAC.

#### Transcription Factors

##### **PRDM16 (PR Domain Containing 16)**

is framed as the gatekeeper of the brown/beige program [62]. It suppresses white at gene networks even while it recruits PGC-1α and CCAAT/enhancer-binding protein β (C/EBPβ) to turn on mitochondrial and thermogenic genes. Without PRDM16, white adipose cells rarely convert into thermogenic beige cells [42, 43, 63]. Its knockdown in subcutaneous white fat, for example, impedes the wound-healing–associated beiging response [64]. Intriguingly, overexpression of PRDM16 (or co‐delivery with partners like FoxP4) via adeno-associated virus (AAV) vectors can provoke weight loss and increase thermogenic gene expression in obese mice, further underscoring its central role [65]. That said, PRDM16 does not act alone or deterministically; its effect is modulated by metabolic context, chromatin state, interacting cofactors and even iron‐dependent signaling in CAC contexts [66]. While PRDM16 promotes beneficial browning in obesity, its aberrant activation in CAC accelerates thermogenic wasting and worsens systemic energy imbalance.

##### **EBF2 (Early B-cell Factor 2)**

specifies thermogenic adipocyte lineage by facilitating enhancer accessibility and recruitment of peroxisome proliferator-activated receptor gamma to brown/beige loci. As a key regulator of metabolic signaling pathways in mature adipocytes [67], it recruits Peroxisome proliferator-activated receptor gamma (PPARy) and reprograms myoblasts or white preadipose cells to brown fat [68, 69]. By reprogramming adipocytes toward a thermogenic lineage, EBF2 could be hijacked by tumors to sustain hypermetabolism.

##### **EWS (Ewing Sarcoma Gene)**

RNA-binding protein was discovered as a crucial regulator of early BAT development. EWS knockout (KO) led to a near-complete loss of embryonic BAT formation, due to incapability of interaction with partner protein Y-box binding protein 1 (YBX1), thereby failed to transactivate and to produce bone morphogenetic proteins (BMP7) [70]. Of note, treatment of EWS null BAT cells with recombinant BMP7 resulted in a full rescue of adipogenic differentiation, indicating that autocrine BMP7 signaling in adipose cells might be important for BAT development [71]. Loss of EWS impairs BAT development, but tumor-driven modulation of BMP7 signaling may alter adipose lineage commitment, influencing CAC progression.

##### **PPARγ**

is known as the master regulator of adipogenesis; but in browning it plays a subtler role: it acts as a molecular hinge between lipid storage and energy burning. PPARγ’s transcriptional outcome depends on which cofactors bind it and on its posttranslational modifications. In browning, it can partner with PRDM16 to drive expression of brown fat–like genes, but in a cell lacking the right epigenome or coactivator milieu it may instead favor white fat pathways [29]. In engineered models, CRISPR activation of *PPARGC1A* or PRDM16 together with PPARγ shifts white adipocytes toward a more thermogenic phenotype [72]. In cancer, PPARγ’s partnership with PRDM16 can amplify browning, shifting adipose tissue toward energy dissipation that contributes to CAC.

##### **C/EBPβ**

 initiates early adipocyte differentiation and activates downstream transcriptional networks required for browning in adipogenesis [73]. C/EBPβ, in coordinating with PPARγ and Placenta-specific 8 (Plac8), drives the thermogenic program, especially responding to acute cold, by switching from white fat-associated C/EBPα to the heat-producing pathway for non-shivering thermogenesis [74, 75]. C/EBPβ mediated cold-induced switch to thermogenic programming may be pathologically activated by tumor signals, fueling adipose wasting.

##### **ATF2 (Activating Transcription Factor 2)**

activates thermogenic gene transcription downstream of p38 mitogen-activated protein kinase signaling during cold or β-adrenergic stimulation. In cancer, it influences cancer progression by activating Wnt/Ca^2+^ signaling pathways and promotes proliferation and invasion in certain types of cancer such as non-small-cell lung cancer [76], indicating that, beyond thermogenesis, ATF2 could regulate tumorigenesis, even probably compounding CAC effects.

##### **CREB (cAMP Response Element–binding Protein)**

mediates transcriptional activation in response to cAMP signaling downstream of β-adrenergic stimulation. In the context of browning, CREB promotes the expression of thermogenic genes, including PGC-1α and UCP1, integrating sympathetic inputs with the transcriptional machinery. Its activity complements ATF2 and other signal-responsive factors, ensuring that environmental cues such as cold or hormonal signals are effectively translated into a thermogenic program [77]. CREB-driven UCP1 induction integrates sympathetic and tumor-derived signals, reinforcing the hypermetabolic state of CAC.

##### **RIP140 (Receptor Interacting Protein 140)**,

also known as nuclear receptor–interacting protein 1 (NRIP1), is a corepressor of genes implicated in glucose metabolism, TriCarboxylic Acid (TCA) cycle, fatty acid oxidation, mitochondrial biogenesis, and oxidative phosphorylation in major metabolic tissues including fat, muscle, liver, and heart [78]. RIP140 acts as molecular silencers of BAT activity and transcriptional (co-)repressor of thermogenic gene expression, mainly through epigenetic or post-translational modifications [79–82]. RIP140 was also shown to block WAT beigeing by preventing the expression of brown fat genes and suppressing a triacylglycerol futile cycle [83]. While repression may be detrimental in obesity by reducing energy expenditure, in cancer contexts it may paradoxically protect against CAC by dampening excessive thermogenesis. Recent work has further highlighted the importance of other transcriptional repressors defined as “thermogenic silencers” in metabolic diseases [84]. However, how do these molecules regulate cellular malignancy and mitigate CAC remain elusive.

#### Transcriptional Co-activators

##### PGC1-α

is cast as the amplifier in this network [85]. Its expression is dynamically responsive to cold and sympathetic signals, and it boosts mitochondrial biogenesis, fatty acid oxidation, and UCP1 transcription directly. Increased PGC1-α correlates with higher thermogenic output in both brown and beige adipocytes [29]. In CAC settings, though, this amplification may become maladaptive: overactive PGC1-α contributes to energy wasting, accelerating fat and lean mass loss [86]. Normally protective, overactive PGC1-α in CAC amplifies mitochondrial biogenesis and energy wasting, accelerating fat and muscle loss.

##### **PGC1-β**

supports sustained mitochondrial function and oxidative capacity in beige and brown adipocytes. Cells deficient in PGC-1b exhibit impaired mitochondrial biogenesis and reduced expression of thermogenesis-related proteins [87]. PGC1-β mediated support of oxidative metabolism may exacerbate tumor-induced hypermetabolism, sustaining CAC progression.

#### Effector Proteins

##### **UCP1**

remains the biochemical signature of thermogenic adipocytes. In the inner mitochondrial membrane, UCP1 dissipates the proton gradient as heat rather than using it for ATP synthesis. This shifts energy toward thermogenesis and away from storage, thus accelerating substrate oxidation [86]. But UCP1 is a double-edged sword: under homeostatic conditions, moderate expression helps regulate body temperature and prevent lipid overload; yet in malignancy, aberrant induction, often triggered by tumor‐derived factors (e.g. PTHrP [45] and catecholamines [88]), can lead to uncontrolled lipolysis and the hypermetabolic state characteristic of cachexia [45, 86, 89, 90]. What complicates matters further is the context dependence: browning in obesity is often beneficial as it raises energy expenditure, dampens inflammation, improves insulin sensitivity, yet once a tumor coopts the thermogenic machinery, the same processes promote tissue loss and worsen patient outcomes [89, 91]. In CAC, excessive UCP1 activity and lipolysis erode both fat and muscle stores, undermining survival and reducing tolerance to therapy [86]. Obesity‐associated adiposopathy, by contrast, fosters a tumor‐friendly niche via chronic inflammation, adipokine imbalance, matrix remodeling and angiogenesis [15, 16]. As a hallmark of thermogenesis, UCP1 is beneficial in obesity but pathologically overexpressed in cancer, driving uncontrolled lipolysis and CAC.

##### **DIO2 (Type II Iodothyroxine Deiodinase)**

enhances local thyroid hormone signaling by converting thyroxine to triodothyronine, thereby amplifying thermogenic capacity. DIO2 is essential for adaptive thermogenesis in brown adipose tissue but shows tissue-specific roles in the control of fatty acid oxidation [92]. By amplifying thyroid hormone signaling, DIO2 enhances thermogenesis, which tumors exploit to intensify systemic energy depletion.

In sum, these transcriptional regulators and effector proteins establish thermogenic lineage, integrate environmental and metabolic cues, balance lipid storage with energy expenditure and amplify mitochondrial biogenesis, oxidative metabolism and uncoupling respiration to execute the browning phenotype. Nevertheless, effective thermogenesis also requires mitochondrial respiratory machinery, lipid catabolism, and glucose uptake pathways, which sustain BAT activity [93, 94]. Beyond the core players described, other factors such as FoxC2 (Forkhead box protein C2), IRF4 (Interferon Regulatory Factor 4), ZFP516 (Zinc Finger Protein 516), and various chromatin remodelers further fine-tune adipose browning process in a context-dependent manner, with layers of redundancy, crosstalk, and tissue-specific modulation [95, 96].

#### Signaling Pathways and Hormone Regulations

BAT activation and development are tightly controlled by environmental cues such as temperature and nutritional status. Central to this regulation is β-adrenergic receptor (β-AR) signaling, which drives both thermogenesis and the differentiation of brown adipocytes. Beyond β-AR signaling pathway, several additional signaling cascades have recently been identified as important contributors to BAT function and recruitment. These pathways act in parallel or downstream of sympathetic inputs, fine-tuning thermogenic capacity and adipocyte phenotype.

##### β-AR Signaling and Hormonal Regulation of Thermogenesis

Norepinephrine released from sympathetic nerves binds to β3-ARs, which are abundantly expressed in BAT and present in WAT [97]. Norepinephrine binding to β‑ARs elevates intracellular cAMP, activating protein kinase A (PKA) and downstream p38 MAPK pathways that culminate in UCP1 induction through phosphorylation of transcriptional regulators including ATF2 and PGC-1α [98]. Among β-AR subtypes, β1-AR is essential for the proliferation of classical brown adipocyte precursors, while β3-AR predominantly controls thermogenic activity in mature brown adipocytes [99, 100]. Notably, β3-AR signaling is dispensable for brown adipocyte development, as β3-AR knockout mice exhibit impaired cold-induced beige cell recruitment without affecting classical BAT development [101]. β3-AR activation initiates downstream signaling cascades that promote thermogenic gene expression, mitochondrial biogenesis, and lipolysis [102]. This stimulation activates brown adipocyte thermogenic function and converts white adipocytes into mitochondria-rich UCP1^+^ beige cells [103]. Cold exposure enhances sympathetic outflow and β3-AR-mediated signaling, resulting in the appearance of UCP1^+^ adipocytes [104]. Continuous sympathetic stimulation maintains this browning phenotype, and inhibition of β3-AR signaling suppresses the expression of brown fat-specific genes in WAT [105]. Adipose browning may occur through multiple mechanisms, including the transdifferentiation of mature white adipocytes into beige cells [104, 106, 107] and the *de novo* differentiation of beige adipocytes from progenitor populations resident in WAT depots [108, 109]. The relative contribution of these pathways appears to depend on physiological context, environmental stimuli, and species‑specific differences [110]. In sum, β-adrenergic signaling represents the central pathway for thermogenic activation as the tumor-derived catecholamines aberrantly activate β-AR pathways and fueling CAC.

In addition to sympathetic inputs, endocrine hormones converge on β‑AR signaling to reinforce thermogenic programming. Exercise‑induced irisin stimulates UCP1 expression and promotes browning of white adipocytes [52, 53], while FGF-21 enhances mitochondrial biogenesis and lipid mobilization [59, 60]. Natriuretic peptides act synergistically with catecholamines to drive thermogenic gene expression and energy expenditure [61]. These hormones, together with β‑adrenergic inputs, integrate environmental and systemic cues to sustain BAT activity. While beneficial in obesity by improving insulin sensitivity and reducing metabolic inflammation, tumor‑derived factors can aberrantly amplify these same pathways, driving uncontrolled browning and energy wasting in CAC. This integration of sympathetic and hormonal regulation underscores the dual role of β‑adrenergic signaling as both a protective metabolic mechanism and a pathological contributor to oncological wasting.

##### **Nitric Oxide (NO) Signaling**

NO activates soluble guanylyl cyclase to generate cGMP, which in turn activates PKG (cGMP-dependent protein kinase). In brown adipocytes, cGMP-PKG signaling induces UCP1 expression and mitochondrial biogenesis [111]. Mechanistically, this pathway enhances PI3K-Akt activity via suppression of RhoA (Ras homolog family member A) and Rho-associated kinase. Beige adipocyte formation in WAT is also promoted by cGMP signaling [112]. However, the physiological relevance of NO signaling in BAT development and thermogenic regulation remains unresolved. While promoting browning under normal conditions, NO signaling may be co-opted by tumors to sustain hypermetabolic states.

##### **PI3K Signaling via PTEN (Phosphatase and TENsin Homolog)**

The PI3K pathway, a downstream effector of insulin and growth factor signaling, is counteracted by the phosphatase PTEN. Unexpectedly, PTEN enhances BAT thermogenic programming by suppressing PI3K activity. Overexpression of PTEN in mouse embryonic fibroblasts promotes their reprogramming into brown adipocytes under the influence of PRDM16 and C/EBPβ [113]. In line with this, pharmacological inhibition of PI3K augments BAT thermogenesis and increases whole-body energy expenditure. In brief, dysregulated PI3K/PTEN balance in cancer alters adipocyte fate, enhancing browning and CAC.

##### **WNTs (Wingless Type MMTV Integration Site Family Members)**

WNT signaling plays a crucial inhibitory role in the regulation of brown adipogenesis. Members of the WNT family, particularly WNT10a and WNT10b, are expressed in BAT but decline as it differentiates, suggesting their negative regulatory role [114]. Overactivation of WNT10b suppresses differentiation by inhibiting the expression of PPARγ and C/EBPα, leading to reduced expression of UCP1 and PGC1-α and impaired mitochondrial biogenesis. As a result, brown adipocytes acquire morphological and molecular characteristics similar to white adipocytes, with diminished thermogenic capacity. Conversely, inhibition of WNT signaling enhances thermogenic gene expression and promotes adipose browning [115]. Suppression of WNT pathways promotes browning, which tumors exploit to drive thermogenic wasting.

##### **mTORC1 (mechanistic Target of Rapamycin Complex 1)**

In adipose tissue, mTORC1 plays a complex role in determining adipocyte phenotype. Its inhibition or loss of raptor in adipocytes promotes adipose browning [116–118], whereas constitutive activation, such as through TSC1 deletion, suppresses brown adipocyte marker expression while increasing white adipocyte markers and lipid accumulation [119]. This shift is accompanied by reduced expression of the brown fat regulator FoxC2 and elevated expression of white fat–associated factors RIP140 and P107 [120]. Altered mTORC1 activity in cancer shifts adipocyte phenotype, contributing to metabolic imbalance and CAC.

##### **TRPV (Transient Receptor Potential Vanilloid)**

Transient receptor potential vanilloid 1 (TRPV1), activated by thermal stimuli and dietary ligands such as capsaicin and capsinoids, stimulates BAT thermogenesis by inducing UCP1 expression [121–124]. In contrast, TRPV4 functions as a negative regulator of oxidative metabolism in adipocytes. Its activation suppresses PGC-1α and UCP1 expression through ERK1/2-dependent signaling, thereby limiting mitochondrial biogenesis and thermogenic programming. Conversely, inhibition or ablation of TRPV4 enhances PGC-1α activity, increases UCP1 expression, promotes adipose browning, and improves energy expenditure and metabolic health [125, 126]. Dietary and thermal stimuli activating TRPV1 may synergize with tumor cues, exacerbating browning and energy loss.

##### **Leptin/Leptin Receptor Pathway**

Leptin is a 16-kDa peptide hormone secreted by adipocytes in proportion to fat stores. In adipocytes, leptin activates AMP-activated protein kinase (AMPK), which phosphorylates and inactivates acetyl-CoA carboxylase (ACC), lowering malonyl-CoA levels and promoting fatty acid oxidation. Concurrently, lipogenic transcription factors (SREBP-1c, ChREBP, FOXO1) and enzymes (fatty acid synthase, stearoyl-CoA desaturase 1, glycerol-3-phosphate acyltransferase) are suppressed, while mitochondrial proteins (cytochrome C oxidase IV, UCP1, UCP2) and the biogenesis regulator PGC-1α are upregulated. This reprograms adipocytes into mitochondria-rich, fat-oxidizing cells, supporting energy expenditure and leanness [127–130]. AMPK activation promotes browning and energy expenditure, but in cancer it can be aberrantly triggered to sustain hypermetabolism and accelerate CAC.

##### **TGF-β (Transforming Growth Factor-β)**

superfamily members, including BMPs and growth differentiation factors (GDFs), play opposing roles in the regulation of brown adipogenesis. BMP7 promotes brown adipocyte differentiation by inducing brown adipogenic regulators such as PRDM16 [71, 131]. Similarly, BMP4 promotes the commitment of mesenchymal cells to the adipocyte lineage and drives beige adipocyte differentiation in subcutaneous WAT [132]. BMP8β enhances BAT thermogenic activity both in mature brown adipocytes and through hypothalamic regulation, though it does not influence differentiation [133]. In contrast, other TGF-β superfamily members such as GDF-8 (myostatin), TGF-β1, and activins suppress brown fat formation and thermogenesis. This inhibition is mediated via Smad3-dependent signaling, as loss of Smad3 enhances beige cell formation and energy expenditure [134]. Blocking TGF-β signaling through neutralizing antibodies or soluble activin receptor constructs increases BAT thermogenesis and confers protection against obesity and insulin resistance [134, 135]. As TGFβ promotes adipose remodeling and fibrosis, and in cancer it can exacerbate browning-associated inflammation, thereby accelerating CAC and supporting tumor progression.

##### **FGF**

-19, FGF-21, and FGF-23 are endocrine members of the FGF family that act through the coreceptors α-Klotho and/or β-Klotho to regulate energy metabolism [136]. Among them, FGF-21 plays a central role in brown and beige adipogenesis. Circulating FGF-21 increases at birth under PPARα activation to stimulate BAT thermogenesis [137]. In adipose tissue, FGF-21 enhances the expression of PGC-1α, thereby promoting the browning of white adipose depots and the differentiation of beige adipocytes [138]. While chronic systemic elevation of FGF-21 can negatively affect bone mass [139], adipose-derived FGF-21 acts locally, offering metabolic benefits without systemic side effects. Thus, local induction of FGF-21 in adipose tissue presents a promising therapeutic approach for obesity, insulin resistance, and related metabolic disorders. In addition, recent study also demonstrated that FGF-6 and FGF-9 induced by exercise and cold could act as autocrine factors to promote UCP1 expression [140]. Elevated FGF-21 enhances browning and thermogenesis, which tumors exploit to intensify systemic energy wasting in CAC.

##### **Glucagon-Like Peptide-1 (GLP-1)**

is an incretin hormone secreted by enteroendocrine L cells in the ileum and colon [141]. It acts through its receptor, GLP-1R, which is expressed in both peripheral tissues and the central nervous system to regulate energy balance. Activation of GLP-1R in the hypothalamus enhances sympathetic nervous system activity, leading to increased BAT thermogenesis and the induction of adipose browning through an AMPK-dependent mechanism [142, 143]. Furthermore, GLP-1R agonists promote adipose browning via SIRT1 activation, linking GLP-1 signaling to mitochondrial biogenesis and enhanced energy expenditure [144]. These mechanisms highlight the role of GLP-1 as a potential therapeutic target for obesity and metabolic regulation. GLP-1R activation enhances browning and thermogenesis, which may be beneficial in obesity but, when dysregulated in malignancy, contributes to hypermetabolism and systemic wasting in CAC.

##### **Glucocorticoids (GCs)**

play a significant regulatory role in brown adipogenesis by exerting an inhibitory effect on thermogenic gene expression. GCs suppress the expression of UCP1 and other brown fat–specific functional genes [145, 146], while inhibition of GC signaling enhances UCP1 expression [147]. Mechanistically, GCs downregulate PRDM16 through the actions of 11β-hydroxysteroid dehydrogenase (11β-HSD) and *miR-27b*, thereby limiting the adipose browning of WAT [148]. Chronic elevated GCs suppresses BAT activity, contributing to metabolic dysfunction and tumor progression.

##### **Irisin**

is a myokine derived from the proteolytic cleavage of the transmembrane protein FNDC5 (Fibronectin type III domain-containing protein 5), whose expression is upregulated by PGC-1α in skeletal muscle. Physical exercise enhances FNDC5 expression and consequently increases circulating irisin levels. Once released, irisin acts on WAT to induce the expression of thermogenic genes, thereby promoting the browning of white adipocytes and increasing overall energy expenditure [47]. The process appears to involve activation of the MAPK p38 and ERK pathways [48], and PPARα has been suggested as a downstream effector. Normally protective via exercise-induced browning, irisin can be aberrantly elevated in cancer, reinforcing hypermetabolic states.

Collectively, these signaling cascades integrate sympathetic, hormonal, and environmental inputs to polish thermogenic capacity.

#### Epigenetic Regulation

##### DNA Methylation and Histone Modification

Chromatin remodeling defines the transcriptional landscape required for brown and beige adipocyte development. Master regulators such as PPARγ are recruited to adipocyte-specific loci together with histone and DNA modifications that activate lineage-selective genes [149, 150]. In pluripotent cells, adipogenic genes (*Pparg*, *Cebpa*) carry bivalent H3K4me3/H3K27me3 marks, which resolve in pre-adipocytes to a poised state with H3K4me3/H3K9me3 [151]. Differentiation signals remove repressive marks, leaving H3K4me3, which allows phosphorylated C/EBPβ and cofactors such as STAT5A (Signal Transducer and Activator of Transcription 5 A) to initiate *Pparg* expression [152].

Subsequently, PPARγ heterodimerizes with RXR and activates adipocyte-specific gene transcription at enhancers enriched with H3K27ac and H3K4me3 [153]. Distinct chromatin states separate BAT and WAT lineages: BAT-selective genes like *Ucp1* are enriched for H3K4me3 and activated in brown adipocytes but are repressed by H3K27me3 in white adipocytes [154]. During brown adipocyte differentiation, H3K27me3 marks on thermogenic genes are progressively removed. *Pparg* remains marked by monovalent H3K4me3 in both BAT and WAT [150, 154].

Beige adipogenesis involves relatively small but critical shifts in PPARγ binding that form beige-selective super-enhancers and accessible chromatin regions [155]. Chromatin conformation further regulates gene expression: long-range looping connects the *FTO* (fat mass and obesity-associated) locus to *IRX3/IRX5*, influencing beige adipogenesis depending on noncoding variants that alter repressor binding [156, 157]. In addition, chromatin remodeling by phosphorylated JMJD1A (Lysine (K)-specific Demethylase 3 A) is required for acute *Ucp1* activation in response to cAMP signaling [158].

##### MicroRNA Regulations

MicroRNAs (miRNAs) are 18–25 nucleotide-long molecules that regulate gene expression by repressing translation or promoting degradation of target mRNAs. They are often tissue-specific, playing key roles in cell differentiation and are implicated in various diseases, including cancer, cardiovascular disorders, and diabetes. Notably, miRNAs fine-tune brown adipogenesis by modulating the stability and translation of target mRNAs [159]. Taking individual miRNA into account for its role in regulating BAT biogenesis, *miR-196a* promotes inducible beige adipogenesis in WAT. Its expression increases upon cold exposure or β3-adrenergic stimulation and enhances beige cell content and energy expenditure when overexpressed. Mechanistically, *miR-196a* targets *Hoxc8*, a white fat determinant that represses *C/ebpβ* through an HDAC3-dependent mechanism. By relieving this repression, the miR-196a–Hoxc8–C/EBPβ pathway promotes beige adopogenesis in WAT progenitors [131, 160, 161]. *miR-133* regulates the myogenic–adipogenic fate decision in satellite cells. Highly expressed in myogenic cells, *miR-133* represses *Prdm16* by binding to its 3′UTR, thereby preventing brown adipocyte differentiation. Knockdown of *miR-133* upregulates PRDM16, leading to UCP1^+^ brown and beige adipocyte formation in muscle and subcutaneous WAT [162, 163]. Overexpression of *miR-133*, conversely, suppresses thermogenic adipocyte development. *miR-155* maintains brown preadipocytes in a proliferative state by repressing differentiation. Overexpression of *miR-155* reduces BAT mass and thermogenesis, while its inhibition enhances UCP1 expression in WAT. This regulation occurs through a bistable negative feedback loop with C/EBPβ: TGFβ1 signaling induces *miR-155*, which represses C/EBPβ translation, whereas C/EBPβ suppresses *miR-155* transcription [164]. Recent studies also identified *miR378* [154], *miR-26* [165], *miR-30* [81]as new positive regulators of the brown and beige fat development, whereas *miR-27* [166], *miR-106b-93* [167], including miR*-155* [168] were identified as negative regulators. However, the comprehensive regulatory network of miRNAs in brown/beige adipogenesis remains to be fully characterized.

In brief, epigenetic and post‑transcriptional mechanisms provide precision control, ensuring browning is context‑dependent and adaptable.

Taken together, adipose browning reflects a multilayered regulatory system: transcription factors and co‑activators establish and amplify thermogenic programs, effector proteins implement heat production, signaling pathways and hormones integrate systemic cues, and epigenetic regulators acclimate gene expression. This interconnected network allows adipose tissue to flexibly shift between energy storage and expenditure, yielding metabolic benefits in obesity but contributing to maladaptive wasting in CAC.

## Dual Effects of Fat Browning in Cancer

The physiological mechanisms underlying the transformation of energy-storing white adipocytes into thermogenic, multilocular beige adipocytes represent a pivotal point of metabolic plasticity with dual effects in oncology. The central effector in this process is UCP1 [3, 86, 169, 170]. While UCP1-mediated thermogenesis is fundamentally beneficial in maintaining systemic metabolic stability and combating obesity by increasing energy expenditure [171, 172], its pathological hyperactivation by tumor-derived factors drives severe catabolic wasting, illustrating its function as a true “double-edged sword” in the context of cancer. Beyond mechanistic insights, several human studies have examined whether BAT activity correlates with cancer incidence or progression. Observational imaging studies using F^18^-FDG (fluorodeoxyglucose) positron-emission tomography (PET) scans have reported heterogeneous associations: in some cohorts, higher BAT activity was linked to reduced metabolic dysfunction and potentially lower oncogenic susceptibility [173, 174]. However, other analyses found no consistent protective effect, underscoring the complexity of BAT’s role in human cancer biology [175].

These findings highlight the translational tension between experimental models, which often demonstrate tumor‑suppressive metabolic benefits of adipose browning, and clinical observations, which remain inconclusive. Integrating this data into mechanistic frameworks emphasizes that BAT’s influence on cancer is context‑dependent, shaped by metabolic state, tumor secretome, and systemic energy balance.

### Modulatory Effects of Adipose Browning on Tumor Progression

In the context of cancer prevention and early-stage disease, the regulated activation of brown and beige adipose tissue functions as an important tumor-suppressive mechanism by mitigating underlying systemic metabolic risk factors. Adaptive browning, often induced by lifestyle factors such as cold exposure or physical exercise, directly antagonizes this pathogenic state [176]. Active BAT may confer a level of direct oncological resilience to the host. Research suggests that chronically ill patients possessing detectable BAT at diagnosis are better equipped to withstand the catabolic stressors of the disease [177]. These individuals demonstrate superior weight maintenance and experience lower risk of developing CAC within the ensuing year [178]. This protective effect may stem from a systemic metabolic advantage or, alternatively, from nutrient competition. The highly active, metabolically demanding BAT may consume shared circulating nutrients, such as glucose and fatty acids, acting like a sink [179], possibly limiting the energy substrates available to fuel aggressive tumor proliferation and growth [180]. Thus, beneficial browning serves not only to prevent metabolic dysfunction but also to bolster host defenses against the metabolic exploitation initiated by tumors.

Adaptive fat browning may also act as a tumor-suppressor by reversing the pro-carcinogenic environment of obesity. Obesity-driven adiposopathy promotes immunosuppressive M2 macrophages and chronic inflammation [181]. Beneficial browning, induced by exercise or cold, improves lipid homeostasis and shifts the immune microenvironment toward M1 predominance, thereby enhancing anti-tumor immunity [182]. In contrast, other evidence indicates that BAT or beige adipose tissue can release factors that promote M2 polarization, which may support tumor growth [183, 184].

These findings underscore the context-dependent nature of BAT’s immunomodulatory effects, with both M1 and M2 polarization pathways potentially engaged depending on tumor microenvironment and systemic metabolic cues. Furthermore, BAT itself is immunologically active, harboring distinct regulatory T-cell populations that contribute to immunometabolism balance [185], offering a new avenue to foster resilience against cancer progression.

Interestingly, a very recent study demonstrated that engineered adipocytes overexpressing UCP1 led to a significant cancer suppression in organoids in vitro and xenografts in vivo [72], implying adipose browning could potentially exhibit anti-cancer impact. Even though emerging accumulating evidence suggests that adipose browning may influence immune signaling pathways, potentially contributing to tumor suppression, current studies remain largely correlative, and the causal sequence linking browning to immune reprogramming and tumor suppression has not yet been convincingly established.

While browning has been associated with changes in cytokine profiles and immune cell recruitment, these findings should be interpreted as preliminary. Further mechanistic investigations are required to determine whether immune modulation represents a direct consequence of browning or a parallel adaptive response. Thus, the role of adipose browning in immune‑mediated tumor suppression should be considered a promising but yet unproven hypothesis.

### Adipose Browning as a Metabolic Amplifier of Tumor-induced Cachexia

While UCP1-related thermogenesis can be beneficial in disease prevention, its excessive activation is frequently observed in CAC models, marked by severe and ongoing loss of both fat and muscle, caused by uncontrolled and persistently high energy use by the body [186]. Current evidence remains largely associative, and direct causal links have not been conclusively demonstrated. Moreover, the tumor itself initiates the pathological adipose browning through the secretion of specific soluble factors called cachexokines, fundamentally hijacking the host’s endogenous adaptive metabolic switch, as seen in cardiac cachexia in colon cancer [187, 188]. These studies were mainly focused on cardiac physiology, and that extrapolation to cachexia is indirect.

This catabolic reprogramming is driven by a complex interplay of systemic and local signals. Among the tumor-derived factors is PTHrP, which promotes the expression of thermogenesis-associated genes in adipose tissue [34]. High circulating levels of PTHrP are commonly found in cachectic patients, correlating positively with increased energy expenditure and reduced lean body mass [189–191]. Other key mediators include the inflammatory cytokine Interleukin-6 (IL-6), which contributes to browning and fat atrophy [192], and factors such as Zinc α2-glycoprotein (ZAG) [193] and growth differentiation factor-15 (GDF-15) [194, 195]. To date, however, there is still no definitive in vivo evidence showing that tumor‑derived cachexokines could directly target browning activity.

The pathological β-adrenergic tone is not solely driven by circulating cachexokines but is locally amplified by a macrophage-sympathetic neuron axis within the WAT [196]. The tumor’s inflammatory environment causes the infiltration and alternative activation of Type 2 (M2) macrophages, which then secrete neurotrophic factors like nerve growth factor (NGF) and brain-derived neurotrophic factor (BDNF) [197]. This local signaling promotes enhanced sympathetic neurite outgrowth and activity, resulting in heightened catecholamine secretion and subsequent β3-AR-mediated hyperlipolysis [198].

The resulting massive release of free fatty acids and other lipids deplete fat stores observed in both animal models [199] and CAC patients. Cancer cells exploit this nutrient abundance for proliferation, creating a vicious cycle where tumor-induced browning supports both cachexia and tumor growth [45, 200]. This sustained catabolic state leads towards the atrophy of fat depots and ultimately, the severe negative energy balance characteristic of cachexia [201].

Inhibition of this pathway, for instance by β3-AR antagonists or PTHrP neutralizing antibodies, has been shown to reduce adipose browning and attenuate cachexia, underscoring that browning might represents a downstream metabolic consequence rather than an independent initiator of carcinogenesis [32, 45, 202]. Taken together, as tumor-derived factors such as PTHrP and IL-6 are considered as primary drivers of CAC, their pathogenic effects could be mediated, at least in part, through the induction of adipose browning [46].

In this context, browning does not directly promote tumor progression but instead functions as a maladaptive amplifier of tumor-induced hypercatabolism. By increasing UCP1 expression and thermogenic activity, browning exacerbates systemic energy wasting, thereby worsening patient morbidity and mortality [199]. Thus, adipose browning should be interpreted as a pathological effector that magnifies the metabolic burden imposed by tumor-secreted signals, rather than as a direct carcinogenic-promoting cue.

## Therapeutics and Public Health Perspectives

The paradoxical role of BAT in both metabolic protection [203] and CAC underscores the need for carefully targeted interventions. Manipulations of thermogenesis associated signaling pathways are explored as potential therapeutic levers to modulate adipose browning. Preclinical studies demonstrate that pharmacological or lifestyle‑based activation of these pathways can improve metabolic health and may attenuate obesity‑related cancer risk. However, in advanced malignancy, the same thermogenic mechanisms risk exacerbating cachexia, highlighting the importance of context‑specific application. Regardless of the unexplored fields of BAT, we describe the available evidence to promote browning of fat for the secondary and tertiary prevention of obesity and other metabolic disorders.

### Lifestyle Interventions in the Browning of Fat

The browning of fat has been previously identified as a potential target to address obesity and obesity-related disorders [204]. Over the past few years, numerous studies have explored the various ways in which the body could promote the browning or beiging of WAT, including cold exposure and exercise, to achieve the thermogenic benefit of BAT. Studies on long-term cold exposure have previously identified the ways or signaling patterns by which this exposure could induce the browning of WAT to promote BAT’s thermogenic activity and improve insulin sensitivity and lipid metabolism [205]. This could be due to batokine signaling including IL6 [206] or FGF 21 [138, 203]. Another modality explored by which the adipose browning is promoted was increasing exercise [60, 204]. However, the duration and intensity of exercise by which browning is promoted are yet to be explored. Lower trends of BAT were previously found in young athletes as compared to non-athletes, contrary to the hypothesized increase in BAT by physical activity [207]. Similarly, a clinical trial did not show exercise-induced changes in BAT volume among sedentary adults [208].

### Brown Fat as a Therapeutic Target: Precision Medicine Approach

The role of brown fats in cancer-related cachexia was previously explored. Various proteins related to adipocyte browning were then explored as potential targets to prevent cancer-related cachexia. Glucose-regulated protein 75 (GRP75) was noted to bind and stabilize adenine nucleotide translocase 2 (ANT2), which leads to the increased expression of UCP1. In vivo, withanone, a GRP75 inhibitor, was noted to reverse this browning and alleviated cachectic phenotypes [209]. Recent preclinical evidence also shows the potential of capsaicin, resveratrol, β3-adrenergic receptor agonists, GLP-1 receptor agonists, and AMPK activators to activate adipose browning [210].

### Preventing Cancer and Cancer-related Cachexia

Current evidence shows the potential of BAT as a target in the prevention of obesity-related cancer and CAC. However, conflicting evidence still prevails. While WAT browning increases UCP1 expression, allowing lipid metabolism in cachectic mice [88], and also increases systemic expenditure contributing to cachexia [91], more recent studies in humans using FDG-PET/CT did not show BAT’s major role in CAC [173, 175]. Rather, the presence of BAT was associated with greater weight maintenance [178].

The lack of conclusive evidence in humans on the role of BAT in metabolic health and cancer-related cachexia needs further investigation in order to inform programs and policies. Moreover, studies have yet to explore how these could be translated into effective strategies affecting public health. This does not preclude the prevention of obesity-related cancer and CAC. Globally, the approach of countries to metabolic health still relies on evidence-based interventions of healthy lifestyle and regular physical activity. This is notable in the key messages of the American Cancer Society [211] and the World Health Organization.

## Conclusion

BAT and adipose browning represent a metabolic paradox in cancer biology, simultaneously offering protection against obesity-driven carcinogenesis while contributing to the wasting syndrome of CAC. The dualistic nature of BAT is governed by context-dependent molecular drivers, including PRDM16, PPARγ, PGC1-α, and UCP1, which can either mitigate metabolic inflammation and enhance insulin sensitivity or, conversely, be co-opted by tumor-derived signals such as PTHrP and IL-6 to fuel hypermetabolism and tissue loss. This review underscores the importance of viewing BAT as a continuum between protection and pathology, where its regulatory mechanisms shape both oncogenic susceptibility and disease progression. Clarifying these pathways is essential for advancing precision therapies that integrate metabolic and oncological perspectives, particularly in regions burdened by both obesity and cachexia. Ultimately, BAT exemplifies the need for nuanced, context-aware strategies that harness its beneficial roles while mitigating its pathological activation in cancer.

An additional and important aspect of BAT is its immune function, including macrophage polarization and cytokine balance. Future research should specifically investigate whether the immune modulation observed in adipose browning represents a causal mechanism of tumor suppression, or whether it reflects a parallel adaptive response, thereby clarifying its translational potential in metabolic‑oncology.

Understanding BAT and the key modulators with respect to cancer can facilitate the shift towards personalized approaches to management. Further, the recognition of depot-specific BAT differences contributes to targeted approaches to treatment. There is also growing interest in modulating browning via B3-adrenergic agents, FGF-21, or cold exposure, and all these require timing and safety studies to translate them into practical and usable technologies.

Finally, obesity and metabolic syndrome remain major contributors to the global cancer burden by driving chronic inflammation and metabolic imbalance. This dual burden is especially critical in LMICs, where obesity-related cancers and malnutrition often coexist. Integrating metabolic monitoring and early cachexia detection into cancer programs, alongside equitable research participation and local capacity building, can help bridge these gaps. Culturally sensitive, community-based interventions are also essential to ensure that advances in BAT and metabolic research lead to more inclusive and sustainable improvements in cancer prevention and care.

## Data Availability

No datasets were generated or analysed during the current study.
